# Spontaneous diaphragmatic hernia with bowel perforation complicated by tension hydropneumothorax

**DOI:** 10.1259/bjrcr.20150465

**Published:** 2016-11-02

**Authors:** Andrea Mardighian, Giulia Roberta Ercolino, Diego Palladino, Giuseppe Guglielmi

**Affiliations:** ^1^Department of Radiology, University of Foggia, Foggia, Italy; ^2^Department of Radiology, Scientific Institute Hospital “Casa Sollievo della Sofferenza”, San Giovanni Rotondo, Italy

## Abstract

In this case report, we describe a rare case of spontaneous diaphragmatic hernia with perforation of the incarcerated ascending colon and subsequent formation of tension pneumothorax. A 73-year-old male with a past medical history of chronic right pleural effusion, restrictive ventilatory impairment and hypertension presented to us for evaluation of severe right chest pain of few days’ duration and severe dyspnoea. The chest radiograph revealed the presence of a loop of bowel in the basal right hemithorax with associated air/fluid levels. A CT scan of the chest confirmed the hydropneumothorax and revealed a right lower lobe ipo-expansion and a flogistic lung consolidation. After surgery, the patient underwent a contrast-enhanced CT scan of the chest, which showed no abnormal findings.

## Background

Diaphragmatic hernia occurs frequently. It is a defect in the diaphragm that allows the abdominal contents to move into the chest cavity. There are several types of diaphragmatic hernias: congenital, extremely rare and due to defective development of the diaphragm’s muscular components; secondary hernia, due to traumas; spontaneous hernia, rare and due to increased intra-abdominal pressure.

In this case report, we describe a rare case of spontaneous diaphragmatic hernia with perforation of the incarcerated ascending colon and subsequent formation of tension pneumothorax.

## Case report

A 73-year-old male with a past medical history of chronic right pleural effusion, restrictive ventilatory impairment and hypertension presented for evaluation of severe right chest pain of few days’ duration and severe dyspnoea.

Upon arrival, he was haemodynamically stable with a rhythmic heart rate of 98 beats per min, blood pressure of 155/70 mmHg and respiratory rate of 38 per min. His oxygen saturation was 94% on room air.

Physical examination showed that the patient was awake and orientated but cyanotic and dyspnoeic. Upon auscultation, it was noted that breath sounds were completely absent in the right hemithorax and there was diffuse inspiratory/expiratory whooping in the contralateral hemithorax. All these physical findings were predictive of pneumothorax and the patient was admitted to the department of radiology for evaluation.

## Radiological findings

Radiological investigations are usually required to make a complete diagnosis, assess the contents of the hernia and evaluate for the presence of any associated abnormality.

**Figure 1.**
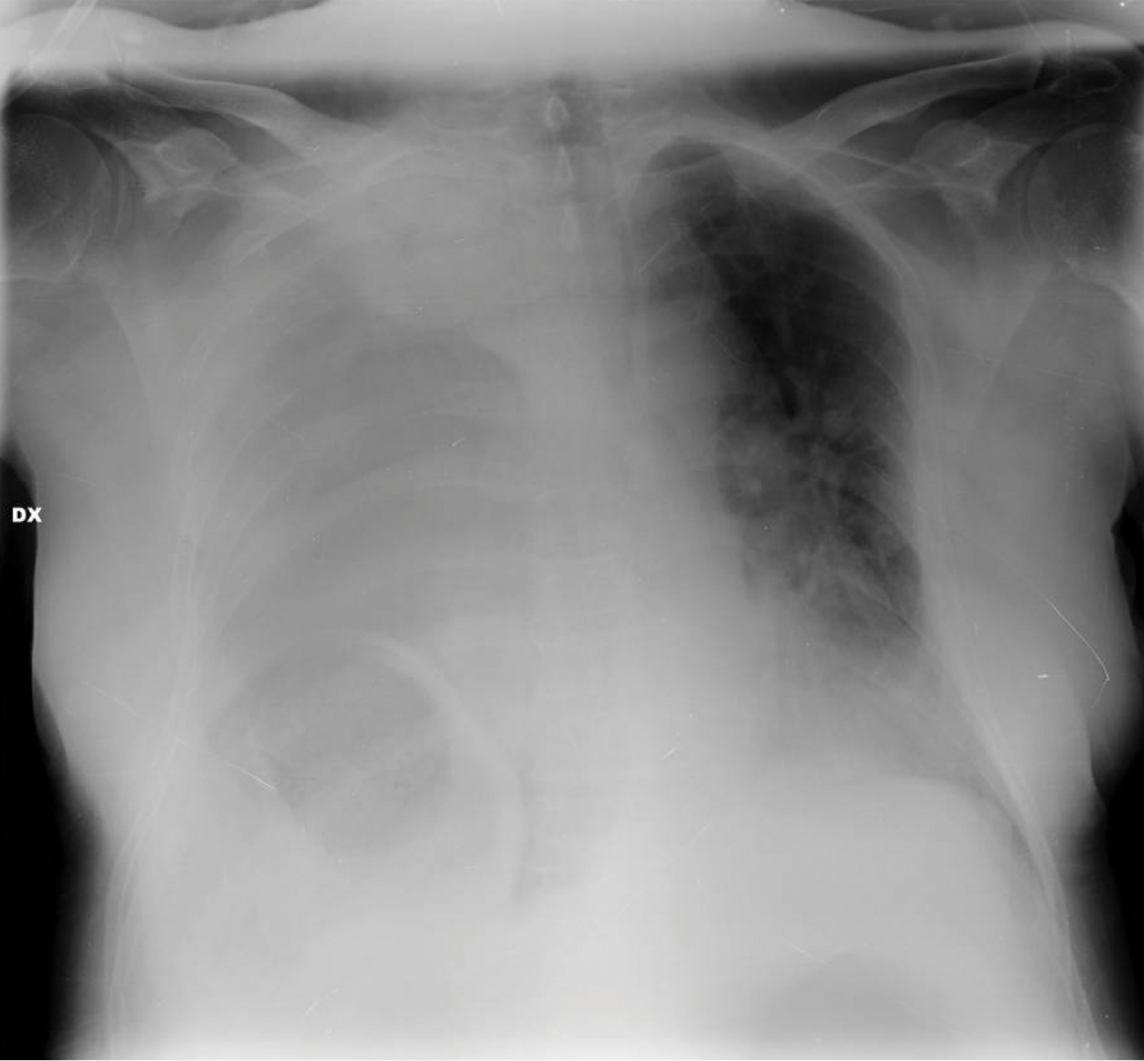
Anteroposterior bedside chest X-ray demonstrating the presence of a diffuse opacity in the right hemithorax.

**Figure 2.**
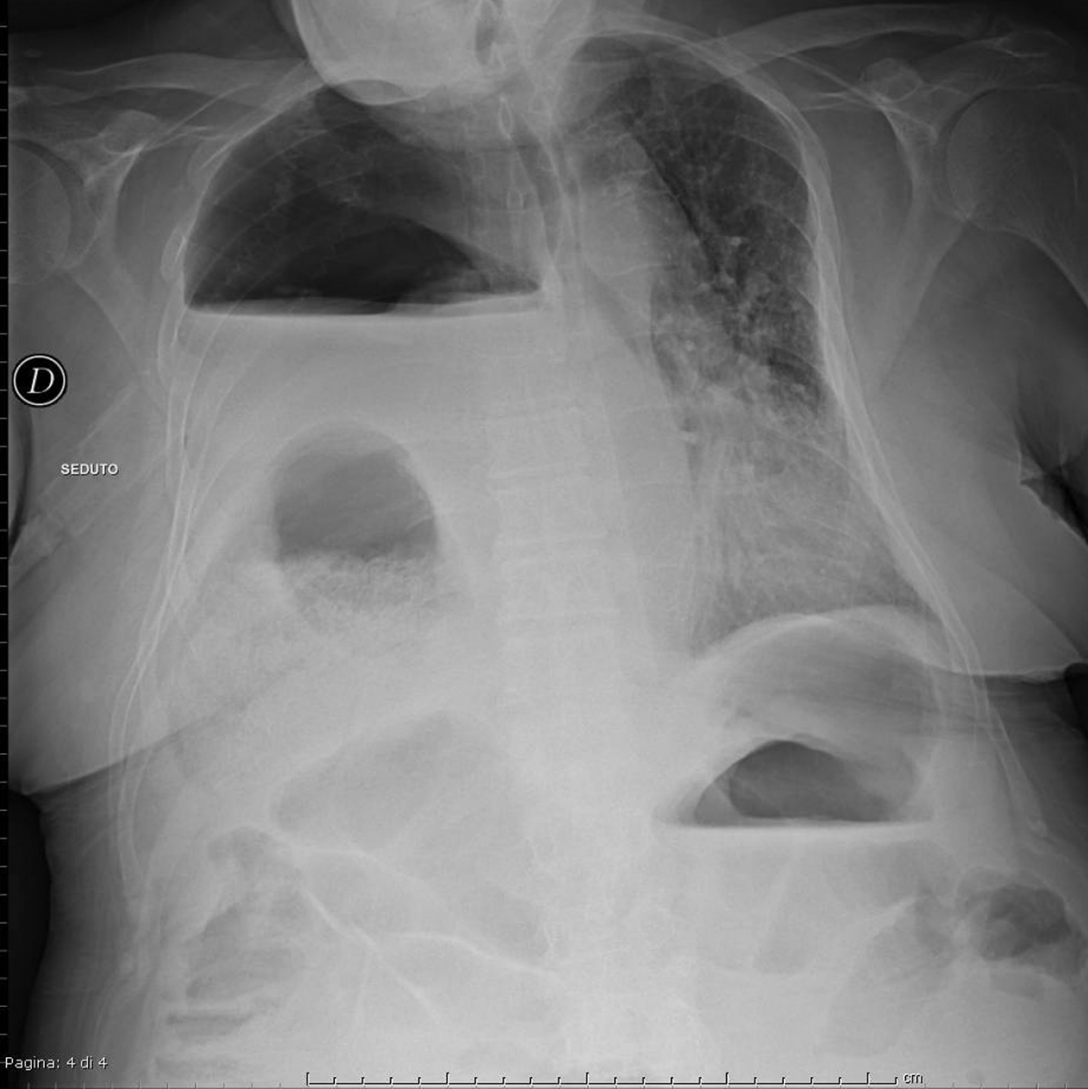
Anteroposterior sitting chest X-ray demonstrating the presence of a bowel loop in the basal right hemithorax with associated air/fluid levels.

**Figure 3.**
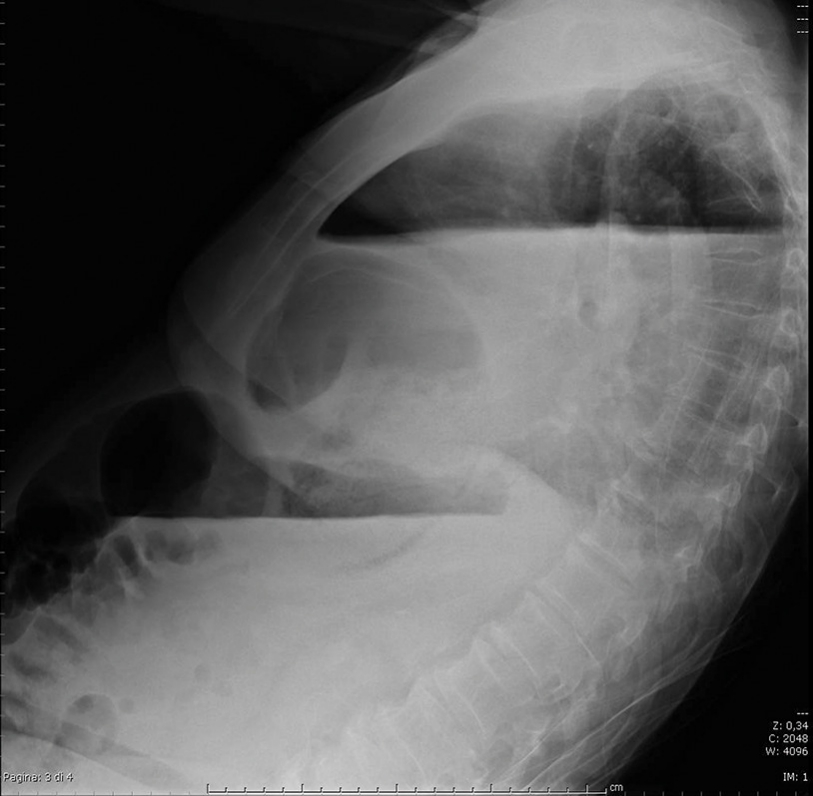
Left lateral sitting chest X-ray demonstrating the presence of a bowel loop in the basal right hemithorax with associated air/fluid levels.

The chest radiograph revealed the presence of a bowel loop at the basal right hemithorax with associated air/fluid levels (Figures 1–3).

**Figure 4.**
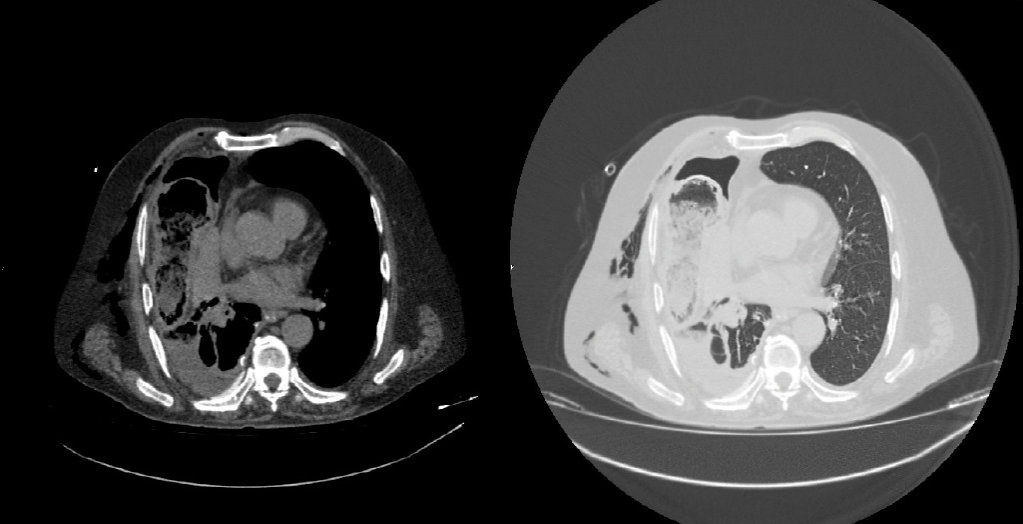
Axial CT scans, before surgery, in both abdomen and lung windows demostrate an ascending colon and its pericolic fat herniation into the right thoracic cavity through a diaphragmatic tear associated with hydropneumothorax, atelectasis of the right lower lobe and subcutaneous emphysema.

**Figure 5.**
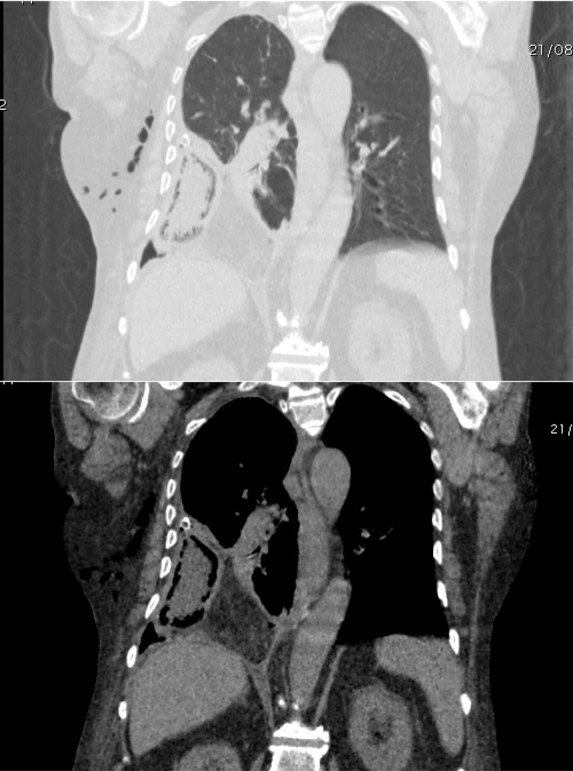
Coronal CT scans, before surgery, in both abdomen and lung windows demostrate an ascending colon and its pericolic fat herniation into the right thoracic cavity through a diaphragmatic tear associated with hydropneumothorax, atelectasis of the right lower lobe and subcutaneous emphysema.

**Figure 6.**
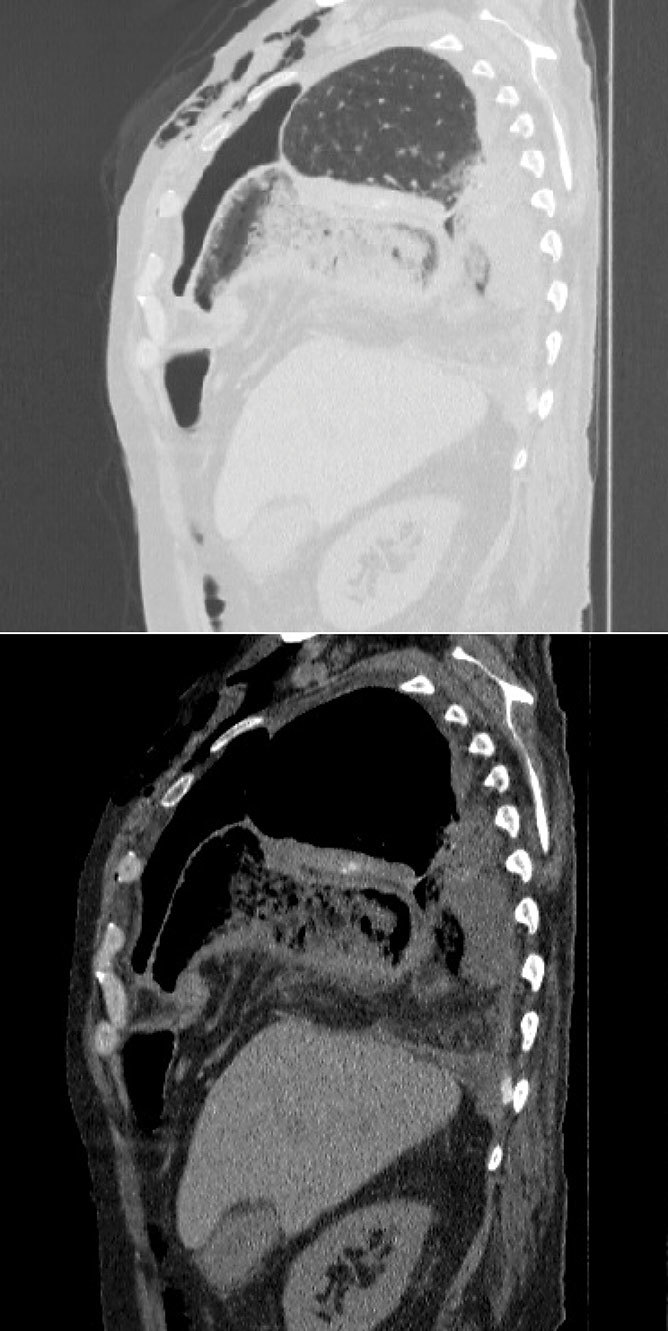
Sagittal CT scans, before surgery, in both abdomen and lung windows demostrate an ascending colon and its pericolic fat herniation into the right thoracic cavity through a diaphragmatic tear associated with hydropneumothorax, atelectasis of the right lower lobe and subcutaneous emphysema.

The chest was then examined by way of a CT scan performed without intravenous contrast because of altered renal values, which showed herniation of the ascending colon into the right thoracic cavity through a 3 cm anterolateral diaphragmatic tear, with bowel wall thickening and ingested materials in the lumen. Furthermore, the pericolic fatty tissue was herniated, with some air bubbles in the pericolic fat. It also confirmed the hydropneumothorax and revealed atelectasis of the lower lobe of the right lung (Figures 4–6).

The findings during surgery confirmed the radiological findings. The patient underwent drainage and disinfection of the pleural space because of the presence of faecal content, and the herniated colon was replaced back in the abdominal cavity after right colon resection with ileocolic anastomosis.

**Figure 7.**
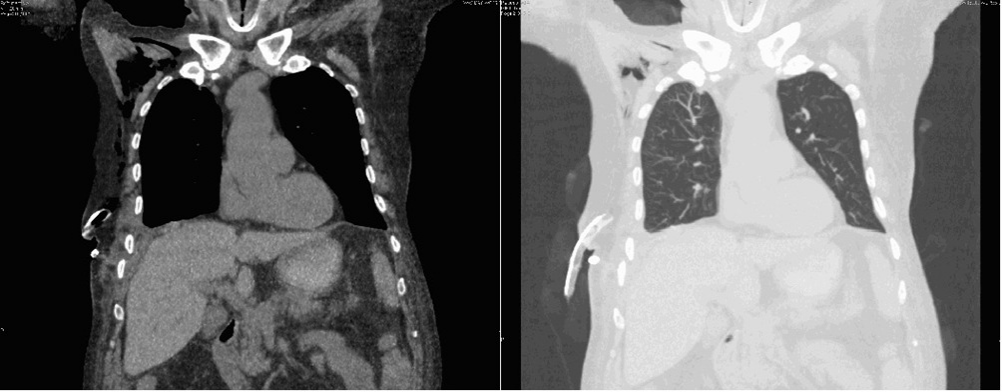
Coronal CT scans, after surgery, in both abdomen and lung windows demostrate successful reduction of the hernia and complete resolution of the hydropneumothorax.

After surgical intervention, the patient underwent a CT scan without intravenous contrast because of altered renal values, which showed no abnormal findings (Figure 7).

## Discussion

Diaphragmatic hernia is often asymptomatic or produces only mild symptoms; however, it may lead to serious complications associated with considerable morbidity and possible fatality, such as intestinal occlusion by incarceration and perforation of the bowel into the thorax, pancreatitis owing to strangled pancreas, rupture of spleen owing to splenic commitment, pneumothorax, superior vena cava syndrome and cardiac arrest.^1^

Spontaneous diaphragmatic hernia is an uncommon entity that accounts for < 1% of diaphragmatic ruptures.^2^ It is usually secondary to conditions that induce an increase in intra-abdominal pressure without any evidence of trauma, such as physical exercises, pilates, vomiting, coughing or delivery.^3^ Although these are the most common causes, our patient did not report any of them.

Two types of spontaneous diaphragmatic hernia are described: Type 1, in which the chest wall remains intact, and Type 2, in which the abdominal structures pass through the chest wall and the diaphragm.^3,4^ Our case fits the description of Type 2 spontaneous incarcerated diaphragmatic hernia with ascending colon perforation, which was complicated by pneumothorax.

The characteristic clinical features of a diaphragmatic hernia are thoracic and abdominal pain, dyspnoea, nausea and vomiting.^5^ The case described here came to us for evaluation of thoracic pain and dyspnoea, probably caused by ascending colon incarceration and pneumothorax.

Pneumothorax is caused by the presence of air in the pleural cavity, “a virtual space” between the lung and the chest wall. There are several types of pneumothorax: spontaneous pneumothorax occurs without any antecedent trauma and can be divided into two types, primary spontaneous pneumothorax, which occurs without any underlying lung disease, and secondary spontaneous pneumothorax, which occurs with underlying lung disease. Traumatic pneumothorax results from penetrating or non-penetrating chest injuries. Our patient was affected by tension pneumothorax. In tension pneumothorax, a “one-way valve” mechanism causes the intrapleural pressure to exceed the atmospheric pressure by allowing air to continuously flow into the lungs but not allowing it to exit, secondary, in our case, to incarcerated bowel perforation through the diaphragmatic hernia.^6^ Furthermore, the pneumothorax was complicated by a past medical history of chronic right pleural effusion.

## Conclusions

Here, we have reported an unusual case of spontaneous diaphragmatic hernia complicated by ascending colon incarceration and perforation and presenting as tension pneumothorax. The cause of the diaphragmatic defect is not clear, although it is unlikely to be a congenital hernia as it was not documented in the past medical and radiological history. The cause of the bowel perforation is also not apparent, as the patient did not have any previous history of endoscopic procedures, such as a colonoscopy, or traumatic injury. The patient underwent surgical intervention, with clinical resolution of his symptoms.

## Final diagnosis

Spontaneous diaphragmatic hernia with bowel incarceration complicated by tension hydropneumothorax.

## Learning points

1. Diaphragmatic hernia is a defect in the diaphragm that allows the abdominal contents to move into the chest cavity.
2. There are several types of diaphragmatic hernia: congenital, extremely rare and due to a defective development of the diaphragm’s muscular components; secondary hernia, due to trauma; spontaneous hernia, rare and due to an increased intra-abdominal pressure.
3. Imaging techniques, such as X-rays, provide valuable clues for possible diagnosis of these lesions, but CT scan is the gold standard.

## Consent

Patient gave informed consent for this specific study.
